# Supplementary material for: Lower Nitrogen Availability Enhances Resistance to Whiteflies in Tomato
Source: Plants (Basel). 2020 Aug 26;9(9):1096. doi: 10.3390/plants9091096 (PMC7569811; doi:10.3390/plants9091096)
Supplement: Supplementary file 1 [file plants-09-01096-s001.pdf]

Supporting information

Authors: Sreedevi Ramachandran, Sylvie Renault, John Markham, Jaime Verdugo, Marta V. Albornoz, Germán Avila-Sakar

Title: Lower nitrogen availability enhances resistance to whiteflies in tomato

Journal: Plants

Appendix A

Literature Review on the Feeding Preference of Herbivores for Nitrogen-rich Plant Tissues

We searched Web of Science using the following parameters: TOPIC (nitrogen herbivory preference); Timespan: 1991-2020. Indexes: SCI-EXPANDED on 25 June 2020, and obtained 198 references. We selected 93 references that contained all three terms “nitrogen” “herbivor” and “preference” in the abstract and then discarded those with “alga”, “aquatic”, or “seagrass” in the abstract. This gave us a list of 72 references, within which we found two studies of aquatic systems and 16 that did not provide information on the relationship between herbivore feeding preference and nitrogen content of plant tissues, leaving us with 54 relevant studies. To this list, we added one reference (Hunt et al. 1994) that we found in one of the studies in our list (Dean et al. 2014), for a total of 55 studies relevant to our question. The spreadsheet document “literature review herbivore preference N.xlsx” contains tables with the references outlined above.

Appendix B

Statistics of the full general linear models (main effects, two-way and, where possible, three-way interactions) mentioned in the main text of the paper.

Table S1. General linear model analysis for the effects of variety and N level on leaf total* chlorophyll content of four tomato varieties grown at three N levels in greenhouse conditions

**Source**	**df**	**Adj. MS**	***F***	***P***
Variety	3	12.319	11.22	0.000
N level	2	12.722	11.58	0.000
Variety*N level	6	1.341	1.22	0.319
Error	36	1.098		
Total	47			

R^2_{adj} = 0.53

Adj. MS: adjusted mean square

* total chlorophyll = chlorophyl *a* + chlorophyll *b*

Table S2. General linear model analysis for the effects of whitefly presence, variety and N level on total fruit fresh weight per plant of four tomato varieties grown at three N levels in greenhouse conditions

**Source**	**d.f.**	**Adj. MS**	***F***	***P***
Whiteflies	1	4113	0.75	0.477
Cage(Whiteflies)	2	5467	3.33	0.038
Variety	3	1244	0.76	0.520
N level	2	33156	20.17	0.000
Variety*N level	6	844	0.51	0.798
Whiteflies*Variety	3	387	0.24	0.872
Whiteflies*N level	2	1405	0.85	0.427
Whiteflies*Variety*N level	6	2338	1.42	0.207
Error	214	1644		
Total	239			

$R^2_{adj} = 0.15$

Adj. MS: adjusted mean square; GLM included variety and N level as fixed effects and whiteflies nested in table as a random effect. The plants were arranged in four cages (two of which had whiteflies) on four tables

Table S3. General linear model analysis for the effects of whitefly presence, variety and N level on total fruit dry weight per plant of four tomato varieties grown at three N levels in greenhouse conditions

**Source**	**d.f.**	**Adj. MS**	***F***	***P***
Whiteflies	1	45.666	1.80	0.312
Cage(Whiteflies)	2	25.436	2.71	0.069
Variety	3	21.459	2.29	0.080
N level	2	179.989	19.17	0.000
Variety*N level	6	15.286	1.63	0.141
Whiteflies*Variety	3	1.169	0.12	0.946
Whiteflies*N level	2	4.733	0.50	0.605
Whiteflies*Variety*N level	6	9.875	1.05	0.393
Error	214	9.387		
Total	239			

R^2_{adj} = 0.17

Adj. MS: adjusted mean square

GLM included variety and N level as fixed effects and whiteflies nested in table as a random effect. The plants were arranged in four cages (two of which had whiteflies) on four tables

Table S4. General linear model for the effects of whitefly presence, variety and N level on individual fruit size as measured by fresh weight in tomato plants of four varieties grown at three N levels in greenhouse conditions

**Source**	**d.f.**	**Adj. MS**	***F***	***P***
Whiteflies	1	0.0262	0.15	0.738*
Cage(Whiteflies)	2	0.1783	0.72	0.490
Variety	3	0.2981	1.20	0.312
N level	2	0.9814	3.94	0.021
Variety*N level	6	0.7590	3.05	0.007
Whiteflies*Variety	3	0.0576	0.23	0.874
Whiteflies*N level	2	0.1956	0.79	0.457
Whiteflies*Variety*N level	6	0.0948	0.38	0.891
Error	203	0.2491		
Total	228			

R^2_{adj} = 0.05

Adj. MS: Adjusted mean squares

Data were log-transformed. Variety and N level were fixed effects and whiteflies were nested in table as a random effect

* not an exact F test

Table S5. Reduced and full general linear model for seed production of four tomato varieties grown at three N levels

Source	d.f.	Adj. MS	*F*	*P*
Whiteflies	1	66.00	1.76	0.316
Cage(Whiteflies)	2	37.61	2.58	0.078
Variety	3	34.94	2.40	0.069
N level	2	46.77	3.21	0.042
Error	231	14.55		
Total	239			

R^2_{adj} = 0.09, Adj. MS: Adjusted mean squares;

Data were square root-transformed; GLM included variety and N level as fixed effects and whiteflies nested in table as a random effect. Two-way and three-way interactions of the factors were removed for clarity. The plants were arranged in four cages (two of which had whiteflies) on four tables

Source	d.f.	Adj. MS	*F*	*P*
Whiteflies	1	66.59	1.70	0.322
Cage(Whiteflies)	2	39.17	2.66	0.072
Variety	3	35.85	2.43	0.066
N level	2	43.43	2.95	0.055
Variety*N level	6	6.31	0.43	0.860
Whiteflies*Variety	3	8.31	0.56	0.639
Whiteflies*N level	2	4.31	0.29	0.747
Whiteflies*Variety*N level	6	22.68	1.54	0.167
Error	214	14.74		
Total	239			

R^2_{adj} = 0.05, Adj. MS: adjusted mean square; Data were square root-transformed for analysis

Table S6. General linear model for the effects of whiteflies, variety and N level on total seed mass produced by plants of four tomato varieties grown at three N levels in greenhouse conditions

**Source**	**df**	**Adj. MS**	***F***	***P***
Whiteflies	1	0.01804	0.39	0.596
Cage(Whiteflies)	2	0.04617	1.58	0.208
Variety	3	0.14656	5.02	0.002
N level	2	0.04183	1.43	0.241
Variety*N level	6	0.03377	1.16	0.331
Whiteflies*Variety	3	0.00699	0.24	0.869
Whiteflies*N level	2	0.02379	0.81	0.444
Whiteflies*Variety*N level	6	0.02281	0.78	0.586
Error	214	0.02921		
Total	239			

R^2_{adj} = 0.04

Table S7. General linear model analysis for the effects of whiteflies, variety and N level on the total vegetative biomass produced by plants of four tomato varieties grown at three N levels in greenhouse conditions

**Source**	**df**	**Adj. MS**	***F***	***P***
Whiteflies	1	37.96	0.99	0.425
Cage(Whiteflies)	2	38.45	1.62	0.201
Variety	3	69.90	2.94	0.034
N level	2	1616.47	68.03	0.000
Variety*N level	6	31.23	1.31	0.252
Whiteflies*Variety	3	27.49	1.16	0.327
Whiteflies*N level	2	28.79	1.21	0.300
Whiteflies*Variety*N level	6	15.24	0.64	0.697
Error	214	23.76		
Total	239			

R^2_{adj} = 0.37

Table S8. General linear model analysis for the effects of whiteflies, variety and N level on root : shoot ratio of four tomato varieties grown at three N levels in greenhouse conditions

**Source**	**df**	**Adj. MS**	***F***	***P***
Whiteflies	1	0.00314	1.07	0.410
Cage(Whiteflies)	2	0.00294	3.06	0.049
Variety	3	0.02631	27.35	0.000
N level	2	0.01368	14.22	0.000
Variety*N level	6	0.00080	0.83	0.546
Whiteflies*Variety	3	0.00041	0.42	0.737
Whiteflies*N level	2	0.00043	0.45	0.637
Whiteflies*Variety*N level	6	0.00074	0.77	0.596
Error	214	0.00096		
Total	239			

$R^2_{adj} = 0.31$

Table S9. Generalized linear model analysis using link logit for the effects of variety, N level and the number of seeds produced per plant on the number of seeds germinated in a growth chamber

**Source**	**d.f.**	**Deviance**	**Residual d.f.**	**Residual Deviance**	**P (>Chi)**
NULL			184	159.96	
Variety	3	16.2909	181	143.66	0.0009884
N level	2	0.2217	179	143.44	0.8950577
Seeds	1	1.4802	178	141.96	0.2237486
Variety*N level	6	9.7996	172	132.16	0.1333469

The call used for the model was:

glm(formula = cbind(germ, non.ger) ~ Variety * N level + Seeds, family = "binomial"(link = "logit"), data = germin)

Table S10. General linear model analysis for the effect of variety and N level on resistance to whiteflies of plants from four tomato varieties grown at three N levels in greenhouse conditions. The model includes the Petri plate used for the assays as a blocking factor

**Source**	**df**	**Adj. MS**	***F***	***P***
Variety	3	5.78	236.5	0.000
N level	2	4.71	288.7	0.000
Variety*N level	6	0.70	14.3	0.000
Plate	19	0.07	0.47	0.972
Error	209	1.70		
Total	239			

$R^2_{adj} = 0.85$

Table S11. General linear model analysis for the effects of variety and N level on compensatory ability of plants of four tomato varieties grown at three N levels in greenhouse conditions and subjected to whitefly infestation

**Source**	**df**	**Adj. MS**	***F***	***P***
Variety	3	32.12	5.526	0.001
N level	2	3.43	0.590	0.556
Variety*N level	6	5.19	0.893	0.503
Error	108			
Total	119			

$R^2_{adj} = 0.14$

Adj. MS: adjusted mean square

Appendix C

References for Table 2

[1] Cadahia, C. 1995. Fertilización del cultivo del tomate. (Coord.: F. Nuez). Ed. Mundi-Prensa. Madrid.

[2] Calderón, S.F. 2002. Requerimientos nutricionales de un cultivo de tomate bajo condiciones de invernadero en la Sabana de Bogotá. Available online: http://www.drcalderonlabs.com/Cultivos/Tomate/Requerimientos_Nutricionales.htm (accessed on 19 August 2020).

[3] Torres, A. 2017. Manual del cultivo de tomate. Boletin Boletín INIA / N° 11, 94 pp. ISSN 0717 – 4829, INIA - INDAP, Santiago de Chile

[4] VanSickle, J., Smith, S., and E. McAvoy, E. 2018 Production Budget for Tomatoes in Southwest Florida. Available online: http://ufdc.ufl.edu/IR00003786/00001 (accessed on 19 August 2020).

[5] Copeval. www.copeval.cl/fertilizantes.html (accessed on 19 August 2020).

[6] Coagra. www.coagra.cl/fertilizantes (accessed on 19 August 2020).

[7] Ficha ODEPA para el cultivo del tomate bajo invernadero. https://www.odepa.gob.cl/fichas_de_costo/fichas_pdf/tomate_invernadero_tutorado_valparaiso_2012-13.pdf (accessed on 19 August 2020)

[8] https://www.indexmundi.com/commodities/?commodity=urea (accessed on 19 August 2020)
